# To: Out-of-bed extubation: a feasibility study

**DOI:** 10.5935/0103-507X.20150049

**Published:** 2015

**Authors:** Amanda Soares Skueresky, Soraia Genebra Ibrahim Forgiarini, Luiz Alberto Forgiarini, Alexandre Simões Dias

**Affiliations:** Course of Physiotherapy, Centro Universitário Metodista - Porto Alegre (RS), Brazil.; Course of Physiotherapy, Universidade Federal do Rio Grande do Sul - Porto Alegre (RS), Brazil.

**To the Editor,**

We congratulate the authors on their article entitled “Out-of-bed extubation: a feasibility study”, which was published in this journal.^(1)^ The issue addressed by this article is extremely important for professionals who work in intensive care units and are looking for paradigm shifts, particularly with respect to the mobilization of chronically critically ill patients.

These patients often experience muscle complications related to immobility. Puthucheary et al. evaluated 63 patients beginning 48 hours after admission to the intensive care unit (at 1, 3, 7 and 10 days) and prospectively demonstrated early changes related to both reduced muscle cross-sectional area and muscle protein metabolism. These changes in muscle, which occur early and rapidly in septic patients, are directly related to increased time on mechanical ventilation.^(2)^

The patient sample evaluated by Dexheimer Neto et al. consisted of septic patients; these patients were compared against patients with other pathologies (including 33 septic patients, 24 patients with cardiac insufficiency, 26 postoperative patients and 10 neurological patients), an approach that introduced bias with respect to heterogeneity.^(1)^

Similar to this investigation by Dexheimer Neto et al.,^(1)^ other studies have reported the effectiveness of early mobilization for seriously ill patients with respect to not only the process of removing these patients from mechanical ventilation but also reducing their length of stay in the intensive care unit. In a systematic review, Li et al. demonstrated that active mobilization protocols for mechanically ventilated patients produce positive in-hospital outcomes; thus, this approach is a safe strategy that can increase muscle strength, providing better conditions for weaning from mechanical ventilation and promoting functional independence.^(3)^

Relative to the new proposal for extubating chronically critically ill patients, the article addressed in this letter appears to utilize a methodological design that limits the assessment of the examined variables.

Thus, this study adds to knowledge regarding the implementation of new strategies for removing chronically critically ill patients from mechanical ventilation and demonstrates the effectiveness of participation by multidisciplinary teams with respect to the therapeutic approaches adopted for patients.^(4)^

However, it behooves us to note that the study results could be enhanced by employing a new methodological design that seeks to randomize the extubation of patients who are either lying in bed or sitting in a chair and thereby avoid potential selection bias.
