# Supplementary material for: To: Out-of-bed extubation: a feasibility study
Source: Rev Bras Ter Intensiva. 2015 Jul-Sep;27(3):293–5. doi: 10.5935/0103-507X.20150049 (PMC4592125; doi:10.5935/0103-507X.20150049)
Supplement: Translation

# Translation: AUTHORS’ RESPONSE

**Authors:** Felippe Leopoldo Dexheimer, Patrini Silveira Vesz, Rafael Viegas Cremonese, Clarissa Garcia Soares Leães, Ana Carolina Tabajara Raupp, Cristiano dos Santos Rodrigues, Juliana Mara Stormovski de Andrade, Raquel da Silva Townsend, Juçara Gasparetto Maccari, Cassiano Teixeira

**Affiliations:** Intensive Care Unit, Hospital Ernesto Dornelles - Porto Alegre (RS), Brazil.; Intensive Care Unit, Hospital Moinhos de Vento, Porto Alegre (RS), Brazil.

Thank you for your compliments and the opportunity to discuss not only the practices of early mobilization and out-of-bed extubation but also the incorporation of these practices into the care of critically ill patients in our country.

First, we note that the benefits of early mobilization (the mobilization of patients who have received mechanical ventilation for less than 48 hours, who compose the population examined in our study) extend to patients with various clinical conditions; in particular, early mobilization can reduce delirium durations, increase patients’ functional abilities at discharge, and decrease lengths of stay in the intensive care unit and in the hospital.^(1^-^3)^

We also agree that for critically ill patients, a diagnosis of sepsis is closely linked to muscle damage and to polyneuromyopathy, a highly prevalent condition among severely ill patients that is detrimental to the process of weaning patients from mechanical ventilation.^(4^,^5)^ However, because the aim of our study was to assess the effectiveness of an unprecedented practice (out-of-bed extubation), we intentionally examined a heterogeneous patient population to ensure that our findings exhibited good external validity.^(6)^

Similarly, a limitation of our study is its retrospective design, which restricts the interpretation of implications related to the safety of this new practice.^(6)^ Once again, we agree that a randomized clinical trial design and better characterization of the patient groups undergoing weaning from ventilation (septic versus non-septic, for example) would provide stronger results; such findings have not yet been published in the literature.

Mechanically ventilated patients benefit from the use of less sedation (through sedation protocols and/or daily interruption) in combination with spontaneous breathing trials and early mobilization.^(7^-^9)^ Because these interventions occur concurrently, we propose that early mobilization does not delay weaning (or *vice versa*); thus, out-of-bed extubation can be regarded as a novel approach, although this treatment still needs to be validated in specific, well-designed studies.^(6)^

In conclusion, we emphasize the importance of communication and the coordination of the efforts of different specialists within a multidisciplinary team (that includes members from medical, nursing, and physiotherapy fields, among others) in attempts to improve the viability of early mobilization combined with concurrent mechanical ventilation.^(3^,^5^,^10^,^11)^

## References

[1] Morris PE, Goad A, Thompson C, Taylor K, Harry B, Passmore L (2008). Early intensive care unit mobility therapy in the treatment of
acute respiratory failure. Crit Care Med.

[2] Schweickert WD, Pohlman MC, Pohlman AS, Nigos C, Pawlik AJ, Esbrook CL (2009). Early physical and occupational therapy in mechanically ventilated,
critically ill patients: a randomised controlled trial. Lancet.

[3] Hodgson CL, Stiller K, Needham DM, Tipping CJ, Harrold M, Baldwin CE (2014). Expert consensus and recommendations on safety criteria for active
mobilization of mechanically ventilated critically ill adults. Crit Care.

[4] Fan E, Cheek F, Chlan L, Gosselink R, Hart N, Herridge MS, Hopkins RO, Hough CL, Kress JP, Latronico N, Moss M, Needham DM, Rich MM, Stevens RD, Wilson KC, Winkelman C, Zochodne DW, Ali NA, ATS Committee on ICU-acquired Weakness in AdultsAmerican Thoracic
Society (2014). An official American Thoracic Society Clinical Practice guideline:
the diagnosis of intensive care unit-acquired weakness in
adults. Am J Respir Crit Care Med.

[5] Schweickert WD, Kress JP (2011). Implementing early mobilization interventions in mechanically
ventilated patients in the ICU. Chest.

[6] Dexheimer FL, Vesz PS, Cremonese RV, Leães CG, Raupp AC, Rodrigues C dos S (2014). Out-of-bed extubation: a feasibility study. Rev Bras Ter Intensiva.

[7] Girard TD, Kress JP, Fuchs BD, Thomason JW, Schweickert WD, Pun BT (2008). Efficacy and safety of a paired sedation and ventilator weaning
protocol for mechanically ventilated patients in intensive care (Awakening and
Breathing Controlled trial): a randomised controlled trial. Lancet.

[8] Kress JP, Pohlman AS, O'Connor MF, Hall JB (2000). Daily interruption of sedative infusions in critically ill patients
undergoing mechanical ventilation. N Engl J Med.

[9] Mehta S, Burry L, Cook D, Fergusson D, Steinberg M, Granton J, Herridge M, Ferguson N, Devlin J, Tanios M, Dodek P, Fowler R, Burns K, Jacka M, Olafson K, Skrobik Y, Hébert P, Sabri E, Meade M, SLEAP Investigators, Canadian Critical Care Trials Group (2012). Daily sedation interruption in mechanically ventilated critically
ill patients cared for with a sedation protocol: a randomized controlled
trial. JAMA.

[10] Nydahl P, Ruhl AP, Bartoszek G, Dubb R, Filipovic S, Flohr HJ (2014). Early mobilization of mechanically ventilated patients: a 1-day
point-prevalence study in Germany. Crit Care Med.

[11] Jolley SE, Regan-Baggs J, Dickson RP, Hough CL (2014). Medical intensive care unit clinician attitudes and perceived
barriers towards early mobilization of critically ill patients: a
cross-sectional survey study. BMC Anesthesiol.
